# Women’s Special Issue Series: Biomedicines (2nd Edition)

**DOI:** 10.3390/biomedicines14071544

**Published:** 2026-07-10

**Authors:** Federica Falà, Riuko Ohashi, Letizia Polito

**Affiliations:** 1 Department of Medical and Surgical Sciences-DIMEC, Alma Mater Studiorum, University of Bologna, 40126 Bologna, Italy; federica.fala@unibo.it; 2 Division of Molecular and Diagnostic Pathology, Graduate School of Medicine, Dentistry, and Health Sciences, Niigata University, Niigata 951-8510, Japan; riuko@med.niigata-u.ac.jp

The second edition of the “Women’s Special Issue Series: Biomedicines” brings together a series of articles that reflect both the scientific excellence of women researchers and the growing complexity of contemporary biomedical research. Although the contributions span diverse disciplines, including oncology, immunology, cardiology and translational therapies, there are several common themes. Specifically, the interplay between inflammation and disease progression, the importance of precision medicine, the identification of novel therapeutic targets, and the development of innovative treatment strategies are recurring concepts throughout the Special Issue.

Inflammation is widely recognized as a hallmark of many chronic diseases, including cancer, where it can both promote tumor progression and contribute to antitumor immune responses, thereby impacting patient survival [1,2,3]. Increasing evidence indicates that several chemotherapeutic agents can induce immunogenic cell death, resulting in inflammasome activation [4,5,6]. The review by Colarusso et al. [7] examined the relationship between conventional chemotherapy, inflammasome activation, and the tumor microenvironment. The authors highlighted the dual role of inflammasome in cancer, which may either enhance antitumor immunity or promote an immunosuppressive tumor microenvironment [8]. They emphasized that the effects of chemotherapy-induced inflammasome activation are highly context-related, depending on tumor type, disease stage, and treatment strategy.

The AIM2 (Absent In Melanoma 2) protein is a member of the innate immunity sensor family that recognizes cytosolic nucleic acids, leading to inflammasome assembly [9]. The biological significance of AIM2 remains controversial because it may function either as a tumor suppressor or as a promoter of tumor progression, depending on tumor histology and cellular context. In a second review, Colarusso et al. provided a comprehensive overview of the current knowledge regarding AIM2 biology and discussed emerging pharmacological approaches aimed at modulating AIM2 activity in different cancer settings [10].

Monoclonal antibodies (mAb) have become the new backbone of clinical practice for many serious conditions, including cancer, Crohn’s disease, psoriasis, and rheumatoid arthritis [11]. However, immunogenicity, defined as the development of anti-drug antibodies (ADA) against these protein-based therapeutics, remains a major clinical challenge [12]. ADA formation can significantly affect the pharmacokinetic profile of therapeutic proteins leading to reduced plasma drug concentrations, diminished therapeutic efficacy, and an elevated risk of adverse events. Advances in protein engineering, in silico epitope prediction, and immune tolerance induction have expanded the toolkit available for mitigating ADA responses [13]. Howard et al. reviewed the mechanisms underlying ADA development and discussed strategies to reduce immunogenicity in mAb therapeutics [14,15]. The authors argued that combining predictive, deimmunization and immunomodulatory strategies may enable the development of safer and more effective biologics with lower immunogenic potential. Particular attention was given to innovative technologies, such as tolerogenic nanoparticles, viral vector-based delivery systems, and nucleic acid-based therapeutic platforms, which represent promising avenues for improving the long-term success of antibody therapies.

Although immunotherapy has revolutionized the management of several malignancies, sex- or gender-related differences in treatment response remain incompletely understood. Biological sex influences disease susceptibility, immune responses and therapeutic efficacy through complex interactions involving genetics, hormones, metabolism, and environmental exposures [16,17,18,19], whereas gender affects health outcomes through identity, social roles, norms and behaviors [20]. The importance of these biological and sociocultural determinants is highlighted by two studies investigating sex-specific differences in clinical outcomes. Calleja-Chucla et al. evaluated the effect of sex on the clinical outcomes of patients with advanced non-small-cell lung cancer treated with immune checkpoint inhibitors [21]. While no significant sex-related differences were observed in overall survival, female patients consistently exhibited significantly shorter progression-free survival than male patients. Buz et al. explored sex-related differences in patients with ST-segment elevation myocardial infarction undergoing successful primary angioplasty [22]. Cardiovascular disease remains the leading cause of mortality worldwide, yet women continue to be underrepresented in many cardiovascular studies [23]. Using advanced echocardiographic parameters, the authors demonstrated significant differences in left ventricular reverse remodeling between men and women, with men demonstrating a more favorable remodeling profile and myocardial recovery.

Growing evidence indicates that chronic stress and emotional regulation influence immune function through neuroendocrine–immune interactions, highlighting a bidirectional neuroimmune communication network whose dysregulation can contribute to immune-mediated disorders [24,25]. Although inflammation is widely recognized as a key factor in breast cancer progression, the underlying biological mechanisms are complex, heterogeneous, and not yet fully understood. Oliveira et al. investigated the inflammatory footprint associated with breast cancer treatments and psychosocial variables in premenopausal women undergoing chemotherapy [26]. The findings confirmed an inflammatory signature, linking the complex interplay between breast cancer, cancer treatment, and psychosocial factors. They support the immunoregulatory role of biological and psychosocial factors in immunocompetence and suggest a new approach focusing on both survival and psychological outcomes.

Gynecological cancers represent a significant global health burden, accounting for approximately 13% of all cancer diagnoses in women worldwide [27]. Several contributions focused on the identification of innovative therapeutic approaches. In a comprehensive systematic review, Ismaili summarized and critically appraised Phase II and III randomized clinical trials and key studies presented at major annual conferences, along with important peer-reviewed publications from 2025 that introduced innovative therapeutic strategies for gynecological malignancies [28]. The review analyzed 23 pivotal clinical trials and identified several new standards of care, emphasizing biomarker-based approaches, the integration of immunotherapy across disease stages, and novel mechanisms to overcome resistance, while also highlighting challenges related to treatment sequencing and global access.

Finally, Biscotti et al. explored the therapeutic potential of plant-derived ribosome-inactivating proteins (RIPs) for colon cancer treatment [29]. RIPs are potent enzymes capable of removing adenines from various polynucleotide substrates [30,31]. RIPs have been widely used in the construction of conjugates and immunotoxins for various medical purposes, showing interesting results, especially in cancer therapy. Saporin is widely regarded as one of the most promising RIPs for the development of targeted immunotoxins [32]. In this study, Sodin 5, a RIP from the edible plant *Salsola soda*, showed high antitumor activity against colon cancer cells, comparable to that of saporin. These findings suggest that Sodin 5 is a promising candidate for future immunotoxin development. More broadly, this study illustrates how natural compounds continue to provide valuable sources of novel therapies for precision oncology.

Taken together, the articles included in this Special Issue illustrate the diversity and interdisciplinary nature of modern biomedical research. While addressing distinct diseases and biological systems, all contributions converge toward a common goal: advancing our understanding of disease mechanisms to enable safer, more effective, and increasingly personalized therapeutic strategies. The studies presented in this Special Issue provide valuable examples of how basic, translational, and clinical research can work synergistically to address complex biomedical challenges.
